# Tracing the Holocene hybrid origin of cultivated walnut in southwestern China

**DOI:** 10.48130/forres-0026-0018

**Published:** 2026-05-15

**Authors:** Jie Liu, Hai-Ling Qi, Peng-Zhen Fan, Chu-Mei Liu, Zeng-Yuan Wu, Ya-Huang Luo, Richard I. Milne, Raees Khan, Lian-Ming Gao, Yue-Hua Wang, Moses C. Wambulwa, Robabeh Shahi Shavvon, De-Zhu Li

**Affiliations:** 1 Germplasm Bank of Wild Species & Yunnan Key Laboratory of Crop Wild Relatives Omics, Kunming Institute of Botany, Chinese Academy of Sciences, Kunming 650201, China; 2 School of Ecology and Environmental Sciences, Yunnan University, Kunming 650091, China; 3 University of the Chinese Academy of Sciences, Beijing 100049, China; 4 Center for Interdisciplinary Biodiversity Research & College of Forestry, Shandong Agricultural University, Tai'an 271018, China; 5 Institute of Molecular Plant Sciences, School of Biological Sciences, University of Edinburgh, Edinburgh EH9 3JH, UK; 6 Department of Life Sciences, School of Science and Computing, South Eastern Kenya University, Kitui 170-90200, Kenya; 7 Department of Biology, Faculty of Science, Yasouj University, Yasouj 74934-75918, Iran

**Keywords:** Cultivated walnut, Chloroplast introgression, Hybridization, *Juglans regia*, *Juglans sigillata*, Southwestern China

## Abstract

Genetic exchanges underpin population adaptation and species evolution. However, knowledge of the genetic impacts of historical, anthropogenic crop-to-wild gene flow remains limited. In southwestern China, the long cultivation and parapatric distribution of *Juglans regia* and *J. sigillata* provide an ideal system to investigate this process, yet the evolutionary origin of the cultivated walnut in this region remains a mystery. Using 31 microsatellite loci to genotype 2,866 individuals, along with 716 chloroplast genomes, we evaluated genetic diversity, population structure, and maternal lineage patterns across the region. Population demographic histories were subsequently inferred using approximate Bayesian computation. Our analysis revealed that *J. sigillata* has higher genetic diversity than *J. regia*, attributed to its long-term persistence in heterogeneous environments. We observed extensive hybridization between the two species around the Sichuan Basin, forming two geographically distinct subgroups: Hybrid1 in the northwest and Hybrid2 in the south. Both subgroups exhibit variable parental contributions, but with the chloroplast genome entirely introgressed from *J. regia*. Furthermore, the Sichuan Basin and Yangtze River acted as major geographic barriers to north-south gene flow. Our divergence time estimates showed that the two species diverged during the Pleistocene, followed by an admixture event between *J. regia* and *J. sigillata*, forming Hybrid2 during the early Holocene. Subsequent admixture between Hybrid2 and *J. regia* produced Hybrid1 during the middle Holocene. We propose that cultivated walnuts in southwestern China originated through introgression between *J. regia* and wild *J. sigillata* during the Holocene, prior to the introduction of agriculture to the region, a process likely facilitated by historical southward human migrations. This research elucidates how long-term human influence has subtly reshaped the genetic landscape of walnuts in southwestern China through gene flow, providing valuable insights for the study and management of other tree crops.

## Introduction

Analysis of the genetic structure, variation, and relatedness within populations can reveal the dynamic history of species and evolutionary relationships of populations^[1,2]^. Insights from such analyses can also help address questions about species origins^[3,4]^ or centers of diversification^[5,6]^. Interspecific hybridization is one of the most significant factors influencing the genetic variation of plant populations, closely connected with the gene flow patterns. In plant populations, gene flow is determined by mating systems, pollinators, population structure, and geographical barriers^[7,8]^, and it can occur between wild and cultivated plants^[9]^. Many domesticated crops, including bananas^[10]^ and certain citrus fruit^[11]^, are believed to have evolved from hybridization between two wild relatives, often accompanied by allopolyploidization^[11−13]^. Previous studies demonstrate the widespread occurrence of gene flow from crops to wild populations in many different species, such as date palms^[14]^, coffee^[15]^, almond^[16]^, apples^[17−20]^, and walnuts^[21−24]^. Nevertheless, the contribution of gene transfer to the origin of cultivated walnut remains unclear.

Common walnut (*J. regia*) and its sister species, the iron walnut (*J. sigillata*)^[25,26]^ are deciduous trees, both diploid with 2*n* = 32^[27,28]^. The two species are ideal for examining genetic exchange and human effects due to their overlapping natural and cultivation ranges in southwestern China. Although *J. regia* is cultivated worldwide, wild *J. sigillata* is narrowly distributed in southwestern China and the Eastern Himalaya^[22,29−32]^. However, it is extensively cultivated within this range, particularly in the Chinese provinces of Yunnan, Sichuan, and Guizhou^[24]^. Hybridization has been observed in much of the *J. sigillata* distribution range (e.g., southwestern China and the Himalaya) where the two species coexist^[22,24,32,33]^. Moreover, it has been demonstrated that human activities play a significant role in shaping the genetic diversity patterns of walnuts in central Asia and the Himalaya^[22,34]^. Therefore, the origins and history of cultivated walnut in southwestern China might have involved repeated selection and adaptation, and/or interspecific gene flow, and hence represents an instructive case of how cultivated tree genotypes arise.

The arrangement of mountains and rivers in southwestern China likely limited agricultural expansion, leading to diverse planting practices and variation between locations during the Late Bronze Age^[35,36]^. The earliest evidence of agriculture in southwestern China is dated around 5,000 years before present (BP), particularly in northwestern Sichuan^[37,38]^. The formation of alluvial terraces and floodplains around 5,500 years BP, possibly triggered by changes to the East Asian Summer Monsoon (EASM), might have contributed to subsequent human settlements in the Sichuan Basin^[39]^. Consistent with this, Gunn et al.^[33]^ posited that *J. regia* and *J. sigillata* remained allopatric until humans introduced *J. regia* from Xinjiang to the Hengduan Mountains. They suggested that human-assisted dispersal could have occurred along key trading routes, such as the Tea-Horse Road along the steep Mekong River canyons in northwestern Yunnan. Long-distance contact networks, oriented along north-south mountain ranges, facilitated not only human migration, but also the exchange of agricultural practices and crops^[40,41]^. The expansion of these networks during the Bronze Age^[42]^ provides a plausible, though not exclusive, framework for understanding how walnuts might have spread alongside human movements. Hence, from this point onward, human-mediated germplasm exchange between introduced species and their wild relatives became increasingly feasible, a mechanism well documented in plants^[43,44]^. Therefore, understanding the genetic history of walnuts in this region can provide insights into broader patterns of agricultural development.

Analyzing molecular characteristics is a key method for deciphering genetic diversity and relationships among walnut species and their cultivars. One of the markers commonly used to assess genetic diversity is Simple Sequence Repeats (SSRs). This marker has proven extremely valuable for elucidating genetic diversity and the relationships between commercially important species such as *J. regia* and *J. sigillata*^[23,32,45]^. More recently, genome sequencing has been employed to elucidate the genetic architecture and evolutionary history of *Juglans* species. Studies based on nuclear and chloroplast genomes have provided insights into the population structure and demographic history of *J. regia* and *J. sigillata*^[22,23,46]^. Nevertheless, constrained by limited sampling across species and geography, these studies remain insufficient to fully clarify the origin and cultivation history of walnut in southwestern China. Furthermore, the anthropogenic dimension of this history remains completely unexamined. In this study, by integrating biparentally inherited microsatellite markers and maternally inherited chloroplast genomes, we utilized a comprehensive genetic dataset in southwestern China to explore: (1) the genetic diversity and population structure of *J. sigillata* and *J. regia* populations, and their genetic relationships; and (2) the mechanism underlying the genetic origin of cultivated walnut. By elucidating genetic patterns within walnut populations in southwestern China, our study provides valuable insights into their origins and guides future conservation and management strategies.

## Materials and methods

### Field sampling

**Figure 1:**
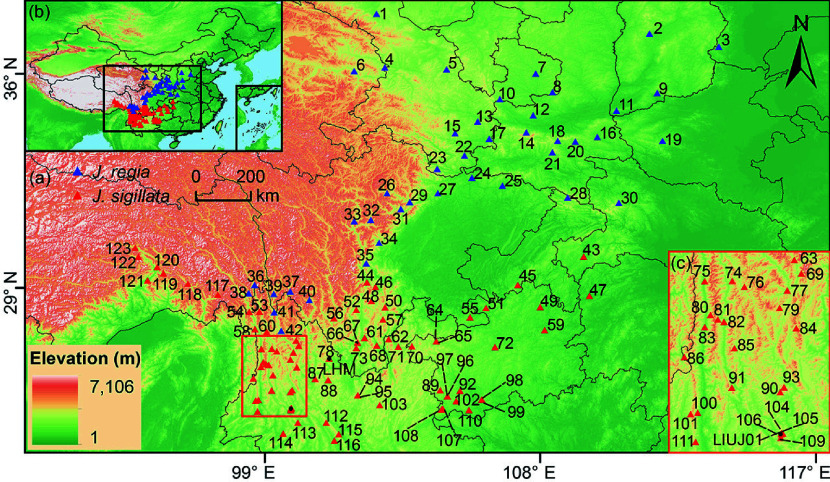
Geographical distribution of 123 populations studied in this study. (a) Sampling sites of *Juglans regia* and *J. sigillata* populations in northern and southwestern China. (b) Geographical location of study region in Asia. (c) Sampling sites of *J. sigillata* in the Three Parallel Rivers region*.* The numbers in the map represent populations IDs (Supplementary Table S1). Blue triangles represent *J. regia* populations; red triangles indicate *J. sigillata* populations.

Based on the walnut distribution data from digital specimens and literature, we conducted field sampling of both *J. regia* and *J. sigillata* in northern and southwestern China between 2013 and 2020 (Fig. 1, Supplementary Table S1). The initial species identification of sampled trees was based on key diagnostic morphological characters. This distinction was primarily based on leaflet number and nut morphology^[33]^. *J. regia* possesses 5–7 leaflets, while *J. sigillata* exhibits 9–17. Furthermore, *J. sigillata* is characterized by pitted nut surfaces and dark kernels with tough septa, contrasting with the wrinkled nut surfaces and light-colored kernels typical of *J. regia*. We collected 5−30 individuals per population, with a minimum distance of > 100 km between populations and > 100 m between individuals (Supplementary Table S1). Additionally, we tried to sample ancient trees with a diameter at breast height (DBH) exceeding 100 cm and an estimated age of over 100 years, as inferred from local knowledge gathered during field surveys. Young leaf materials were collected and immediately dried using silica gel. In total, 2,866 samples from 123 populations (42 *J. regia* and 81 *J. sigillata*) were collected across 13 provinces in China. These included data previously published^[22,32,47]^ (detailed collection information is provided in Supplementary Table S1). In addition, a total of 716 chloroplast genomes were used in our study (Supplementary Table S2), including 204 newly generated in this work, and 512 assembled from previous studies^[23,48]^.

### DNA extraction, PCR amplification, microsatellite genotyping, and sequencing

Approximately 0.20 g of silica gel-dried leaf material was used for DNA extraction following a modified CTAB method^[49,50]^. Subsequently, DNA quality and concentration were assessed using 1% agarose gel electrophoresis and a Nano Drop® ND-2000 spectrophotometer (Thermo Fisher Scientific, Wilmington, DE, USA). DNA samples meeting PCR quality standards were diluted to a concentration of 30−50 ng/μL and stored at −20 °C for further use.

Based on our previous research^[32,51,52]^, a total of 31 microsatellite loci (Supplementary Table S3) were used to genotype the samples. PCR amplification was performed using a multiplex PCR system on a Veriti® 96-well Thermo-Cycler (Applied Biosystems, Foster City, California, USA), following the protocol developed by Xiahou et al.^[52]^. PCR products were verified by 1% agarose gel electrophoresis, and fragment sizes for each individual were determined using an ABI3730xl DNA analyzer (Applied Biosystems, CA, USA). Genotyping data were processed using GeneMarker v2.2.0 (SoftGenetics, State College, PA, USA) and formatted for downstream analyses using GenAIEx v6.51b2^[53]^.

For newly generated chloroplast genomes, genomic DNA was fragmented into ~500 bp inserts for library preparation using the NEBNext Ultra II DNA Library Prep Kit for Illumina (New England BioLabs, Massachusetts, USA), following the manufacturer's instructions. Sequencing was performed on the DNBSEQ-T7 platform (BGI, Shenzhen, China), producing 150 bp paired-end reads. For each individual, approximately 20× coverage was obtained, yielding over 12 Gb of high-quality, clean data.

### Microsatellite data analysis

#### Clonal individuals and Hardy–Weinberg equilibrium

Genetic homogeneity among clones can bias diversity estimates by artificially lowering metrics such as expected heterozygosity while simultaneously inflating others, like observed heterozygosity^[54−56]^, thereby confounding inferences of population structure^[57]^. Consequently, we first used GenAIEx 6.51b2 to define clonal individuals as those with identical genotypes across all 31 loci examined. Additionally, clustering analysis (see the 'Population inference structure' section below) was used to check for unusual groupings among the 2,866 individuals. In the case of anomalies, the individuals were visually checked and re-genotyped whenever necessary. Furthermore, Arlequin v3.5.2^[58]^ was used to estimate whether populations were in Hardy–Weinberg equilibrium.

#### Genetic diversity estimation

Genetic diversity parameters for loci, populations, and different groups were calculated using GenAIEx. These parameters comprised the number of alleles (*N*_A_), the effective number of alleles (*N*_E_), observed heterozygosity (*H*_O_), expected heterozygosity (*H*_E_), unbiased expected heterozygosity (*uH*_E_), and the fixation index (*F*). Additionally, polymorphism information content (*PIC*) was calculated to characterize the genetic polymorphism of loci using the PIC_CALC software (https://github.com/luansheng/PIC_CALC). For genetic diversity at population and group levels, the total number of alleles (*N*_T_) and the number of private alleles (*N*_P_) were also calculated using GenAIEx, while the allelic richness (*A*_R_) was calculated using the *hierfstat* package in R (https://cran.r-project.org/web/packages/hierfstat). Inbreeding coefficients (*F*_IS_) for populations and groups were calculated using FSTAT v2.9.4 (www2.unil.ch/popgen/softwares/fstat.htm) (Table 1).

**Table 1:** Genetic diversity within three groups based on microsatellite data. JR: *Juglans regia*; JS: *J. sigillata*.

Group	*N*	*N*_T_	*N*_P_	*N*_A_	*N*_E_	*H*_O_	*H*_E_	*uH*_E_	*A*_R_	*F*_IS_
JR	871	164	3	5.29	1.94	0.37	0.42	0.42	6.00	0.12
JS	821	237	36	7.65	2.62	0.51	0.58	0.58	8.16	0.13
Hybrid	1,011	207	5	6.68	2.67	0.51	0.58	0.58	7.37	0.11
Total	2,703	251	−	8.10	2.88	0.46	0.61	0.61	8.05	0.24
*N*, sample size; *N*_T_, total number of alleles; *N*_P_, private alleles; *N*_A_, number of alleles; *N*_E_, effective number of alleles; *H*_O_, observed heterozygosity; *H*_E_, expected heterozygosity; *uH*_E_, unbiased expected heterozygosity; *A*_R_, allelic richness; *F*_IS_, inbreeding coefficient.										

#### Population structure inference

Genetic differentiation coefficients (*F*_ST_) between populations and groups were calculated using Arlequin. Standard genetic distances *D*_A_ between populations and groups were calculated using MSA v4.05^[59]^. Analysis of molecular variance (AMOVA) was conducted using GenAIEx for all populations and groups.

Bayesian clustering analysis was performed using STRUCTURE v2.3.4^[60]^. The number of groups (*K*) was set from 1 to 10, with 100,000 burn-in iterations and 1,200,000 MCMC replications after burn-in, repeated 20 times for each *K*. The optimal *K* value was determined using STRUCTURE HARVESTER v0.6.1^[61]^, and repeated sampling analysis was performed using CLUMPP v1.1.2^[62]^. The results were visualized using DISTRUCT v1.1^[63]^.

Based on the admixture coefficient (*Q*) calculated from STRUCTURE analysis at *K* = 2, individuals were classified into pure groups if they had a *Q* value ≥ 0.8, while individuals with a *Q* value 0.2 ≤ *Q* < 0.8 were assigned as hybrids^[22]^. This genetics-based classification scheme was employed to provide an objective baseline assessment of admixture, independent of morphological traits that may be influenced by environmental factors and phenotypic plasticity. It therefore serves as an unbiased reference framework for comparison with the phenotypic identification. Ultimately, this classification resulted in three groups: pure *J. sigillata*, pure *J. regia*, and hybrids. To infer relatedness among all individuals, principal coordinates analysis (PCoA) based on genetic distance *D*_A_ was performed using GenAIEx, and visualized with R package *ggplot2* in R^[64]^. A Neighbor-joining (NJ) analysis was conducted using Populations v1.2.31^[65]^, and the resulting clustering tree was graphically represented using the R package *ggtree*^[66]^. A neighbor-net diagram was constructed using SplitsTree v4.18.3^[67]^ to visualize the genetic relationships among all individuals.

#### Spatial genetic patterns, Mantel test, and barrier analyses

To illustrate the spatial distribution of genetic structure among populations, STRUCTURE results at *K* = 2 were mapped using ArcMap v10.7 (ESRI, Redlands, CA, USA). Additionally, interpolation analysis was performed using inverse distance weighting (IDW) in ArcMap v10.7 to visualize the geographic distribution of genetic diversity parameters, such as allelic richness (*A*_R_) and expected heterozygosity (*H*_E_).

We performed Mantel tests using the mantel function in the R package vegan^[68]^ to assess the patterns of Isolation by Distance (IBD). To distinguish broad-scale, landscape-level patterns from those specific to major genetic groups, we employed a two-level approach. First, we conducted a global analysis of all 123 populations, correlating pairwise genetic differentiation (*F*_ST_) with geographic distance (km) and testing the relationships between genetic diversity (*H*_E_) and altitude, longitude, and latitude using the Pearson correlation. Subsequently, group-level analyses were performed separately for *J. regia* (JR), *J. sigillata* (JS), and hybrid populations. This stratified approach is essential because a significant global signal can be driven by a single group; separate tests allow us to identify whether IBD or environmental correlations operate consistently across the genetic landscape or are group-specific.

To analyze isolation barriers, we employed Monmonier's maximum-difference algorithm in Barrier v2.2^[69]^, a method effective for identifying sharp genetic discontinuities that may correspond to barriers to gene flow. For this analysis, we used the Pairwise genetic distance matrices (*D*_A_) generated from 100 bootstrap replicates in Microsatellite Analyzer (MSA), which were then integrated with the geographical coordinates (Supplementary Table S1). The algorithm initially connects the geographic coordinates through Delaunay triangulation, followed by generation of the corresponding Voronoi tessellations. Barriers were outlined based on these tessellations, and the robustness of the identified boundaries was assessed using Monmonier's maximum difference algorithm. While absolute genetic distance values depend on the specific metric used, the relative spatial positions of identified barriers and the overall pattern are robust to the choice of distance measure. Potential geographical barriers from the Barrier analysis were subsequently displayed in space using ArcMap v10.7.

#### Inference of demographic history

**Figure 2:**
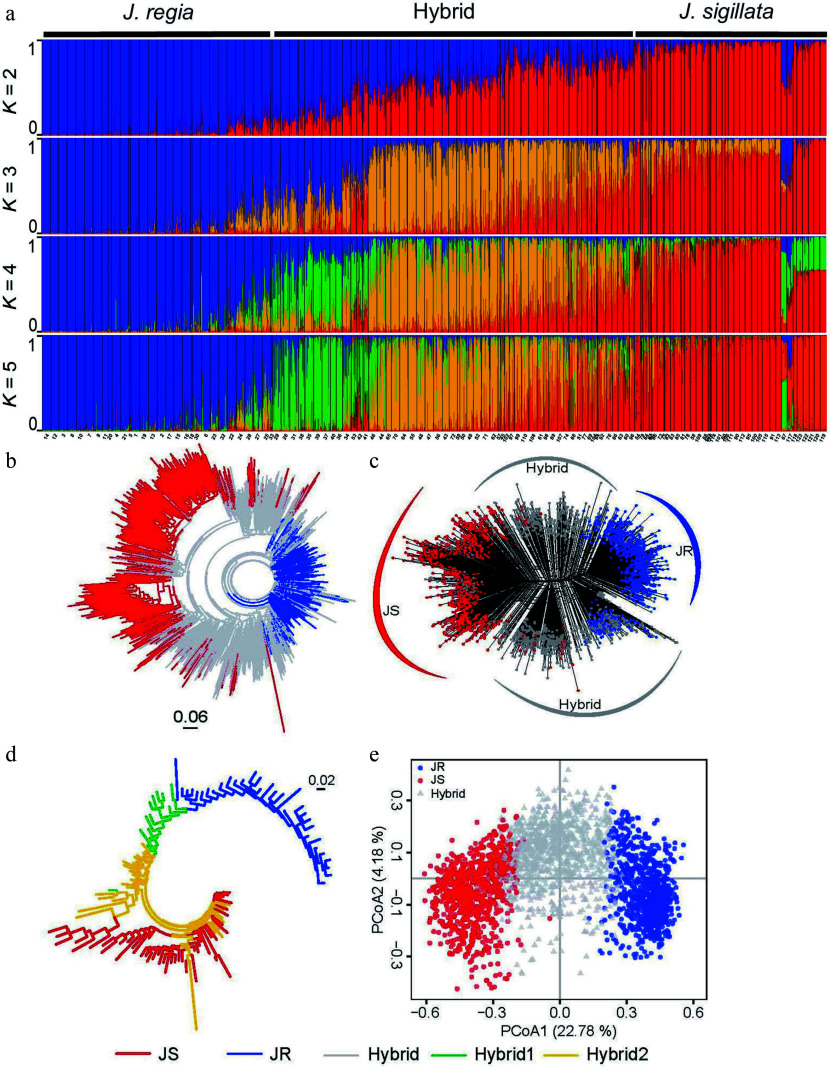
Population structure of 2,703 walnut individuals (*Juglans regia* and *J. sigillata*) based on 31 microsatellite loci. In all five diagrams, various colors indicate distinct genetic groups: blue for JR (*J. regia*), red for JS (*J. sigillata*), grey for Hybrid, green for Hybrid1, and yellow for Hybrid2. (a) STRUCTURE results for *K = *2 to *K = *5. (b) Neighbor-joining tree, and (c) neighbor-net tree of all individuals based on Nei's genetic distance (*D*_A_). (d) Neighbor-joining tree for 123 populations based on Nei's genetic distance. (e) Principal coordinates analysis (PCoA) of all individuals based on *D*_A_ genetic distance.

We employed the approximate Bayesian computation (ABC) approach using supervised machine learning in DIYABC-RF v1.2.1^[70]^ to explore the demographic history of the genetic groups identified by STRUCTURE at *K* = 2. For this analysis, the hybrid group was further divided into Hybrid1 and Hybrid2, which were treated separately. We developed five plausible demographic scenarios, as illustrated in Fig. 2. All parameters, except admixture and divergence time, were set to default values (Supplementary Table S4), with the generation time assumed to be 15 years^[48]^. For model calibration, we used a mean microsatellite mutation rate of 5 × 10^−4^ mutations per locus per generation (a standard value widely used in population genetic studies of eukaryotes^[71−73]^. This allowed us to convert the scaled time parameters estimated by DIYABC-RF into absolute times in generations, which were then converted into years using the generation time. While the absolute timing of events relies on the chosen mutation rate and generation time, the sequence of those events, for instance, whether divergence came before admixture, is derived from the model's fit to the genetic data. This relative ordering remains stable under reasonable adjustments to these calibration values. We generated approximately 2,000 simulations per scenario for scenario choice, and 10,000 simulations for parameter estimation. The choice of scenario and estimation of the posterior probability of the best-supported scenario was performed using the Random Forest algorithm module within DIYABC-RF, which produced 2,000 Random Forest trees for each analysis^[70]^. Additionally, we performed Bayesian analysis of historical migration rates with Migrate-n 5.0.4^[74]^. One long chain was run, retaining 50,000 sampled generations at intervals of 100 generations after a burn-in period of 1,000,000 generations.

### Chloroplast genome data analysis

A total of 716 individuals of *J. regia* and *J. sigillata* were newly assembled and analyzed for their chloroplast genomes, including 204 that were sequenced in this study. The remaining 512 individuals, comprising 460 from Ji et al.^[48]^ and 52 from Ding et al.^[23]^, with project numbers PRJNA721107 and PRJNA356989, respectively, were downloaded from the National Center for Biotechnology Information (NCBI; www.ncbi.nlm.nih.gov) using the SRA Toolkit v2.10.8^[75]^. The chloroplast genomes were assembled and annotated using GetOrganelle v1.7.7.0^[76]^, with the *J. regia* chloroplast genome^[22]^ serving as the reference sequence. The chloroplast genomes were initially aligned using Geneious v9.0.2^[77]^. The resulting alignment files were imported into R, where haplotypes were identified using the *haplotypes* package v1.1.2^[78]^. The identified haplotype sequences were then aligned using Geneious v9.0.2. A haplotype network was constructed using PopART v1.7^[79]^, and a geographical distribution map of the chloroplast genome haplotypes was created using QGIS 3.44.0^[80]^.

Phylogenetic relationships among haplotypes were inferred using Maximum Likelihood (ML) in IQ-TREE (v2.3.5)^[81]^ and Bayesian Inference (BI) in MrBayes v3.2.7a^[82]^. The following seven chloroplast genomes were downloaded from NCBI and used as outgroups owing to their close phylogenetic relationship with the *Juglans*: *Pterocarya stenoptera* (MN262640), *Cyclocarya* *paliurus* (MW118603), *Platycarya* *strobilacea* (MH189595), *Annamocarya* *sinensis* (MN911165), *Carya* *cathayensis* (MW410227), *Engelhardia* *fenzelii* (MT991009), and *Rhoiptelea* *chiliantha* (NC_053773). The resulting trees were visualized using iTOL (https://itol.embl.de). To infer relatedness among haplotypes, principal components analysis (PCA) was carried out using the R package *adegenet* v2.1.8^[83]^, and visualized with the R package *ggplot2*.

### Maternal lineage bias assessment

Given that the separation of pure *J. regia* and *J. sigillata* individuals was consistently supported by both nuclear and chloroplast data, the maternal parent of each hybrid individual was inferred through phylogenetic analysis of chloroplast genome haplotypes. Consequently, hybrid individuals were unambiguously classified as originating from either *J. regia* or *J. sigillata*. Among the 1,011 individuals identified as hybrids based on microsatellite data, we counted the number possessing the *J. regia* and *J. sigillata* chloroplast genome haplotype. The proportion of hybrids with maternal *J. regia* lineage was calculated along with its 95% confidence interval (using the Wilson score method). To statistically evaluate whether the maternal contribution was symmetric, we tested the observed proportion against a null expectation of 0.5 using a two-sided exact binomial test.

## Results

### Microsatellite data

#### Clonal individuals and Hardy–Weinberg equilibrium

Out of the 2,866 individuals screened, 163 were identified as clones. After removing these clonal individuals, the final microsatellite dataset comprised 2,703 individuals from 123 populations. Genetic diversity results for 31 microsatellite loci are provided in Supplementary Table S5. The number of alleles (*N*_A_) across marker loci ranged from 4 (in JS28 and JR07) to 13 (in JS22). The mean effective number of alleles (*N*_E_), expected heterozygosity (*H*_E_), and observed heterozygosity (*H*_O_) were 2.88, 0.61, and 0.46, respectively. Polymorphic information content (*PIC*) ranged from 017 (in JM5446) to 0.76 (in BFU-Jr38), with a mean of 0.55. (Supplementary Table S5). The Hardy–Weinberg equilibrium test indicated that 93.97% of the population–locus combinations conformed to Hardy–Weinberg expectations. Accordingly, the complete dataset comprising 2,703 individuals (Supplementary Table S6) was retained for downstream analyses.

#### Genetic diversity of populations and groups

The number of total alleles (*N*_T_) per population ranged from 55 to 127, and private alleles per population ranged from 0 to 4. The mean number of alleles (*N*_A_) and effective number of alleles (*N*_E_) across the 123 populations were 3.21 and 2.12, respectively. The mean expected heterozygosity (*H*_E_) and allelic richness (*A*_R_) were 0.46 and 1.99, respectively. Additionally, 39 populations had *F*_IS_ values below 0, which may indicate heterozygote excess relative to Hardy–Weinberg expectations (Supplementary Table S1).

Based on the results of genetic structure (Fig. 2a), all individuals were categorized into three groups: JR (*J. regia*), JS (*J. sigillata*), and a Hybrid group (all admixture individuals). For genetic diversity analyses, hybrids were treated as a single group, as these metrics are not designed to distinguish between potentially different hybrid origins. Among the three groups, JS had higher diversity than JR for all variables calculated (Table 1). For example, JS had the highest number of alleles (237) and private alleles (36), compared to 164 and three in the JR group. Additionally, the JS and Hybrid groups showed higher values of observed heterozygosity (*H*_O_), expected heterozygosity (*H*_E_), and allelic richness (*A*_R_) compared to the JR group.

#### Genetic structure and its spatial patterns

The analysis of 2,703 individuals across 123 populations revealed a significant drop in Delta *K* values after *K* = 2, indicating that *K* = 2 was the optimal value. Based on the set *Q* value threshold, the 2,703 individuals were divided into three groups: JS (*J. sigillat*a, 821 individuals), JR (*J. regia*, 871 individuals), and the Hybrid groups (1,011 individuals). Specifically, 871 individuals were assigned to the JR group, 821 individuals to the JS group, and 1,011 individuals to the Hybrid group (Fig. 2a, Supplementary Table S7). This finding was further supported by the STRUCTURE results at *K*3-*K*5 (Fig. 2a), which not only revealed extensive admixture between the JR and JS groups, but also identified subgroups within the Hybrid group, designated as Hybrid1 (green) and Hybrid2 (yellow).

Phylogenetic analyses based on the Neighbor-joining (NJ) and Neighbor-net trees were generally consistent with STRUCTURE results, clearly delineating the three genetic groups (Fig. 2b, c). Additionally, the Neighbor-joining tree revealed that the Hybrid group was split into Hybrid1 and Hybrid2 at the population level (Fig. 2d). The PCoA analysis also yielded similar results, further supporting the clustering of individuals and populations into three different groups (JR, JS, and Hybrid groups), with the first two axes together explaining 26.96% of the total genetic variation (PCoA1: 22.78%, PCoA2: 4.18%). Significant differences among groups were observed only along the PCoA1 axis, with the JS and JR groups clustering on opposite sides, while the Hybrid group clustered between them (Fig. 2e). Therefore, the PCoA, NJ, and Neighbor-net analyses all corroborated the STRUCTURE results, confirming the clustering of individuals into two main groups (JR and JS) and one Hybrid group, comprising two subgroups (Hybrid1 and Hybrid2).

Overall, the pairwise genetic differentiation (*F*_ST_) results for the 123 populations indicated moderate genetic differentiation (Supplementary Table S8, Supplementary Fig. S1), ranging from −0.04 between CYM and LYM (both *J. sigillata*) to 0.63 between FGT (*J. sigillata*) and XJR (*J. regia*). Additionally, pairwise comparative analysis of genetic distance (*D*_A_) showed a minimum of 0.02 between GQR and HSR, SGR and TBR, XXR and TBR, JCR and WQR, SGR and JCR (all *J. regia*), and a maximum of 0.64 between TBR (*J. regia*) and MMR (*J. sigillata*) (Supplementary Table S8, Supplementary Fig. S1).

Analysis of Molecular Variance (AMOVA) at different hierarchical scales revealed distinct patterns of genetic variation partitioning and genetic differentiation (Table 2). When all populations were analyzed without prior grouping, the majority of genetic variation (64%) was found within populations, while variation among populations accounted for 36% (*F*_ST_ = 0.379, *p* = 0.001). When AMOVA was done separately for the three groups, 71% of the genetic variation was within groups and 29% among groups (*F*_ST_ = 0.290, *p* = 0.001). However, when the two hybrid subgroups (Hybrid1 and Hybrid2) were further distinguished, the proportion of variation among groups decreased to 18%, accompanied by a reduction in genetic differentiation (*F*_ST_ = 0.183, *p* = 0.001). Additionally, genetic differentiation between the JR and JS groups is high (*F*_ST_ = 0.464, *p* = 0.001)., with nearly half of the total genetic variation partitioned 46% between them, compared to within groups. Furthermore, the JS-Hybrid comparison shows 16% genetic variation between groups (84% within groups; *F*_ST_ = 0.163, *p* = 0.001), and the JR-Hybrid comparison shows 21% variation between groups (79% within groups; *F*_ST_ = 0.215, *p* = 0.001).

**Table 2:** Analysis of molecular variance (AMOVA) of 123 populations and three groups of *Juglans regia* and *J. sigillata*.

Scale	Source	d.f.	Sum of squares	Mean squares	Percentage of variation (%)
	Among pops	122	24,768.26	203.02	36
	Within pops	2,580	38,593.24	14.96	64
Total	Total	2,702	63,361.50		100
	*F*_ST_	0.37*			
	Among groups	2	13,572.47	6,786.24	29
	Within groups	2,700	49,789.03	18.44	71
Groups	Total	2,702	63,361.50		100
(JS/JR/ Hybrid)	*F*_ST_	0.29*			
JS/JR/ Hybrid1/ Hybrid2	Among groups	3	7,182.079	2,394.026	18
	Within groups	2,699	43,865.67	8.12	82
	Total	2,702	51,047.749		100
	*F*_ST_	0.18*			
	Among groups	1	12,759.23	12,759.23	46
	Within groups	1,690	29,482.96	17.45	54
JS/JR	Total	1,691	42,242.19		100
	*F*_ST_	0.46*			
	Among groups	1	3,599.82	3,599.82	16
	Within groups	1,830	37,035.60	20.24	84
JS/Hybrid	Total	1,831	40,635.42		100
	*F*_ST_	0.16*			
	Among groups	1	4,518.02	4,518.02	21
	Within groups	1,880	33,059.49	17.58	79
JR/Hybrid	Total	1,881	37,577.51		100
	*F*_ST_	0.21*			
d.f., degree of freedom. * *p* = 0.001.					

**Figure 3:**
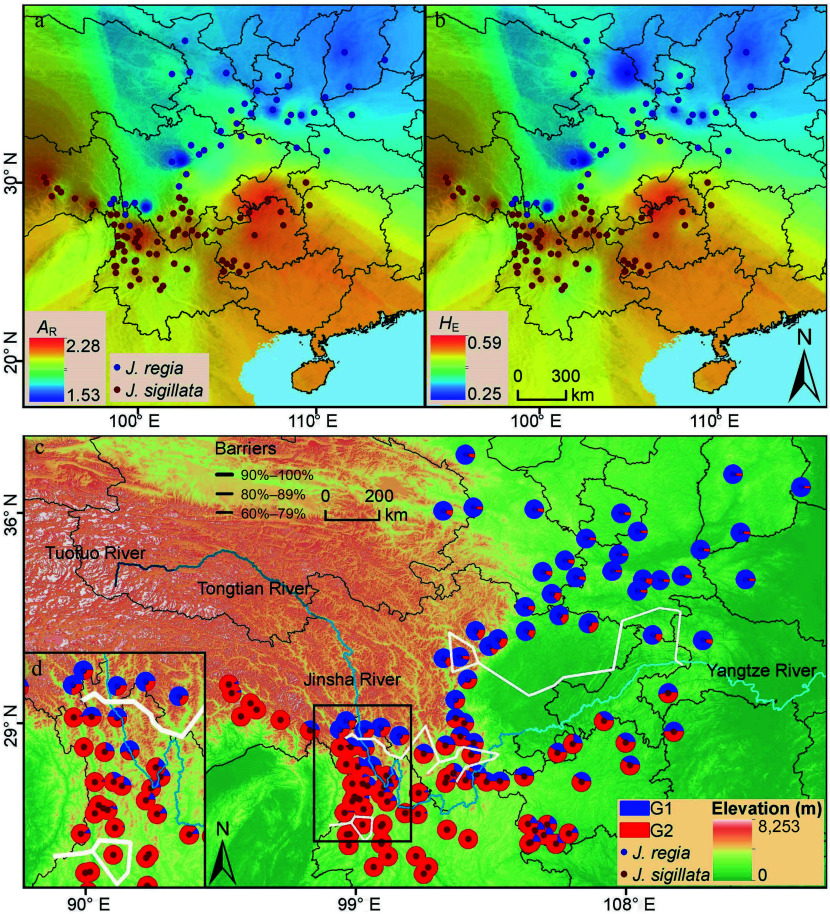
Spatial patterns of genetic diversity and population structure in 123 walnut populations. Genetic diversity was mapped using IDW (Inverse distance weighted) interpolation in ArcMap: (a) allelic richness (*A*_R_); (b) expected heterozygosity (*H*_E_); (c) geographical distribution of the population structure. The map is based on the results of STRUCTURE at *K* = 2. (d) The inset shows the population from the Three Parallel Rivers region. The solid, thick white lines indicate the locations of the two most likely barriers identified through the Barrier analysis.

Spatial mapping of the three genetic groups indicated distinct geographic ranges for the JR and JS groups, with the JR group primarily distributed north of the Sichuan Basin and the JS group mainly distributed in southeastern Tibet and western Yunnan (Fig. 3c). The Hybrid group occupied regions adjacent to the JR and JS groups, such as the Sichuan Basin, eastern Yunnan, and Guizhou. Regarding the distribution of the Hybrid group, hybrids in the northwestern Sichuan Basin and the upper Three Parallel Rivers region were dominated by the JR genetic component. Conversely, hybrids in other parts of Yunnan, the southeastern Sichuan Basin, and Guizhou were primarily composed of the JS genetic component. Consequently, these subgroups were distinguished as Hybrid1 and Hybrid2, respectively, which exhibited distinct geographic distributions. Hybrid1 populations occurred primarily on the northwestern periphery of the Sichuan Basin, whereas Hybrid2 was predominantly found in cultivated stands to the south. This spatial separation suggests divergent origins. Genetically identified pure *J. sigillata* (JS) accessions corresponded closely to wild populations. In contrast, the Hybrid2 group consistently consisted of cultivated populations known locally as soft-shell walnut (Chinese: 绵核桃, Mian Hetao). These results indicate that much of the material cultivated in this region under the name *J. sigillata* is in fact of hybrid origin.

Genetic diversity (*A*_R_, *H*_E_) interpolation for the 123 populations suggested that *J. sigillata* populations have higher genetic diversity compared to the *J. regia* and Hybrid populations. Notably, high genetic diversity of *J. sigillata* was observed in Yunnan, Guizhou, and adjacent regions in southwestern China, which correspond to lower latitudes, while populations at the northern edge (higher latitudes) of the species range exhibited low genetic diversity (Fig. 3a, b, Table 1, Supplementary Table S7).

The Mantel test revealed a significant positive correlation between the geographical distance and genetic distance across populations (*r* = 0.698, *p* = 0.000) (Supplementary Fig. S4a). At the group level, the JR (*r* = 0.595, *p* = 0.000) and JS (*r* = 0.611, *p* = 0.000) groups exhibited significantly higher values than the Hybrid group (*r* = 0.191, *p* = 0.014) (Supplementary Fig. S4b−S4d). These results indicate that isolation by distance is stronger in the parental species than in hybrids, consistent with the expectation that admixture can weaken spatial genetic structure. Moreover, the analysis of the relationship between genetic diversity (*H*_E_) and latitude demonstrated a moderately negative correlation (*r* = −0.481, *p* = 1e−04), suggesting that genetic diversity decreased with increasing latitude. In contrast, correlations between genetic diversity (*H*_E_) and both altitude and longitude were weakly negative but statistically significant (*r* = −0.105, *p* = 0.004; *r* = −0.155, *p* = 3e−04, respectively) (Supplementary Fig. S2).

The Barriers analysis identified two distinct geographical barriers, both with a strength of 99% (Fig. 3c). One barrier was located in the Sichuan Basin and Yangtze River, separating the *J. regia* and *J. sigillata* populations, and dividing the Hybrid group into two subgroups (i.e., Hybrid1 and Hybrid2). The second geographic barrier was located in the Three Parallel Rivers region, effectively separating the northern *J. regia* and southern *J. sigillata* populations. Although different distance metrics or analytical parameters could slightly alter the precise positions of the identified barriers, the overall spatial patterns remain robust. Consequently, the identified barriers provide strong geographical corroboration for the genetic structure of the three groups (JR, JS, and Hybrid) inferred across the study region (Fig. 3c).

#### Inference of demographic history

**Figure 4:**
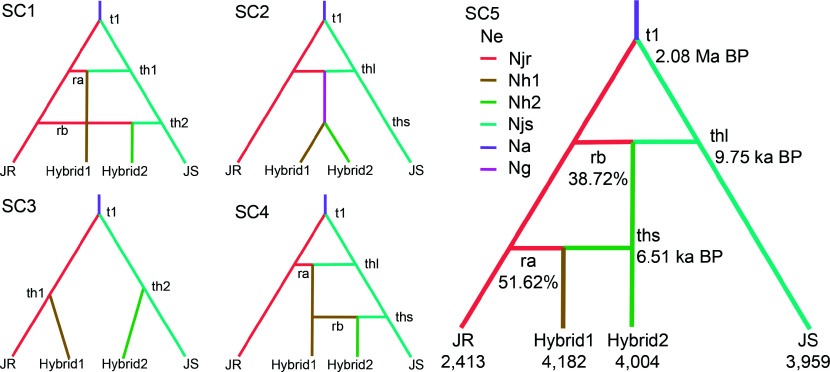
Population demographic history of walnuts inferred from DIYABC-RF analysis. Demographic and historical parameters include six effective population sizes (Njr, Nh1, Nh2, Njs, Na, and Ng) and five events representing population divergence or admixture (t1, th1, th2, thl, and ths). For the scenarios with admixture, the parameters ra and rb represent the genetic contribution of each of the source groups. Branch colors indicate discrete effective population sizes in the model, with time measured in generations, and is not calibrated to scale. The descriptions of model parameters are provided in Supplementary Table S4.

While previous analyses treated hybrids as a single contemporary group, our demographic model explicitly distinguishes between two hybrid subgroups (Hybrid1 and Hybrid2) to model distinct temporal origins and differing parental contributions. DIYABC-RF analyses showed that the best fit model was scenario 5 (976/2,000 votes), with a posterior probability of 0.61 (Fig. 4, Supplementary Table S4). This model indicated that JS split from JR around 2.08 Ma BP (95% CI: 0.84–5.29 Ma BP), followed by an admixture event between JR and JS forming Hybrid2 around 9.75 ka BP (95% CI: 2.75–14.61 ka BP), with subsequent admixture between Hybrid2 and JR producing Hybrid1 around 6.51 ka BP (95% CI: 1.49–14.45 ka BP). The effective population sizes of JR, Hybrid1, Hybrid2, and JS were 2,413 (95% CI: 826–4,080), 4,182 (95% CI: 752–9,180), 4,004 (95% CI: 930–8,629), and 3,959 (95% CI: 1,438–6,636), respectively. Historical migration rates inferred from Migrate revealed relatively symmetrical historical migration rates between groups (Supplementary Table S9). The highest migration rate was observed from Hybrid2 to Hybrid1 (0.83; 95% CI: 0.61–1.04), whereas the lowest rate was detected from Hybrid1 to JS (0.72; 95% CI: 0.50–0.92).

### Chloroplast genome

#### Genome data acquisition and assembly

A total of 204 individuals were newly sequenced, and 512 additional individuals were successfully downloaded from the NCBI database, resulting in 716 whole genome sequences in total. The sequencing depth of the downloaded whole-genome sequences ranged from 11× to 91×, averaging 25×. After assembly and annotation, 716 chloroplast genomes were successfully obtained (Supplementary Table S2).

#### Haplotype clustering and phylogenetic analyses

**Figure 5:**
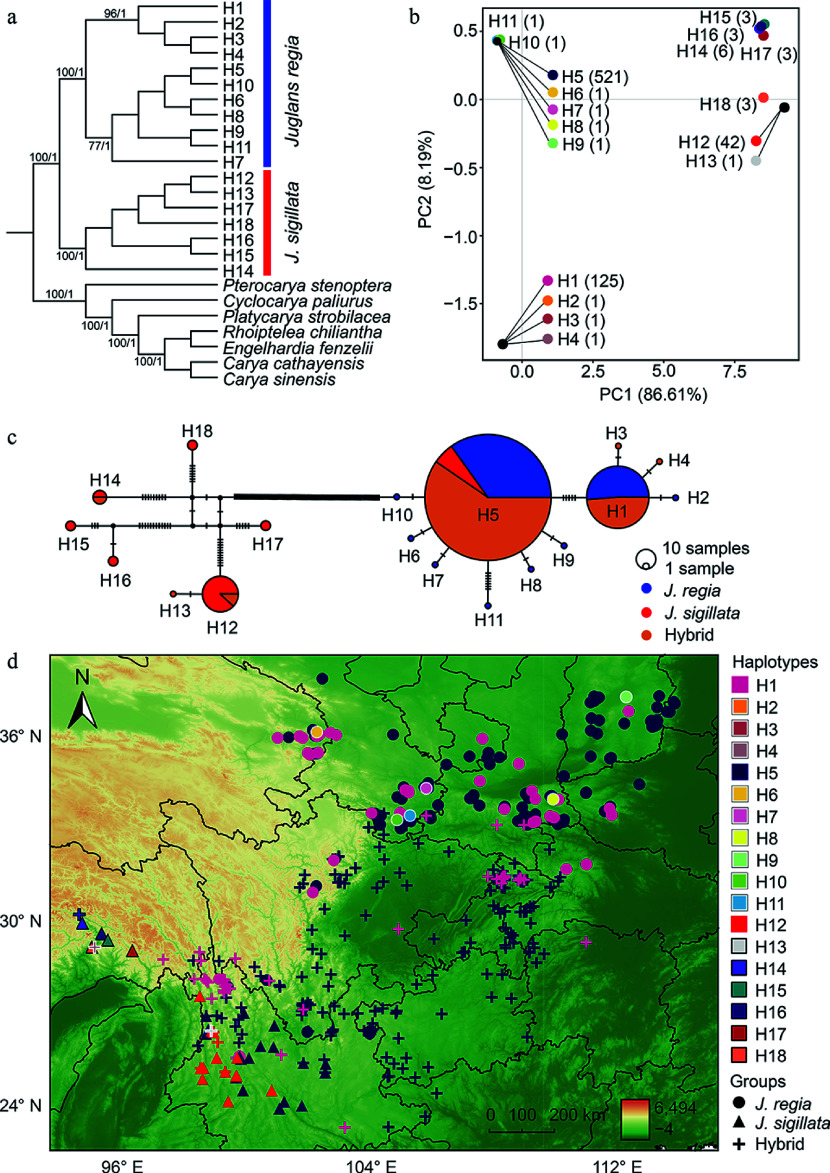
Clustering relationships and geographical distribution of chloroplast genome haplotypes. (a) Phylogenetic tree inferred using maximum likelihood and Bayesian inference. Numbers on the branches indicate maximum likelihood bootstrap supports (BS) and Bayesian posterior probabilities (PP), respectively. Only major clades and values greater than 70 are shown. (b) Principal component analysis (PCA) of 18 haplotypes identified from 716 chloroplast genomes. The numbers next to the haplotypes indicate the number of individuals carrying each specific haplotype. Haplotypes that overlap due to small genetic distances are indicated by external lines. (c) Network of 18 haplotypes based on 716 chloroplast genomes. The size of each circle corresponds to the proportion of the specific haplotype. Vertical lines indicate mutational steps between haplotypes. (d) Geographical distribution of 18 haplotypes in 716 walnut individuals. Private haplotypes were indicated with white and black outlines for *J. regia* and *J. sigillata*, respectively. Different colors correspond to distinct haplotypes, while different shapes represent *J. regia* (circle), *J. sigillata* (triangle), and hybrids (plus sign).

Eighteen haplotypes (H1–H18) were identified across the 716 chloroplast genomes (Supplementary Table S2). Network analysis revealed two major clades: one dominated by 11 *J. regia* haplotypes (H1−H11) and another by seven *J. sigillata* haplotypes (H12–H18) (Fig. 5c). Among these, H1 and H5 were widespread, while H3, H4, and H6−H11 were private in *J. regia* (Fig. 5c, Supplementary Table S2). Despite 92 mutation steps between the two clades, the presence of haplotypes from both species among individuals identified as hybrids is consistent with past hybridization. The star-shaped pattern observed in the *J. regia* clade is compatible with a recent population expansion. Moreover, multiple haplotypes (H1, H2, H5, H12, and H14) were shared across *J. regia* and *J. sigillata*, patterns that may reflect historical introgression and/or incomplete lineage sorting. Specifically, the widespread occurrence of haplotype H5 not only across its own species but also in *J. sigillata* individuals and hybrids, together with the population structure revealed by microsatellite data (Fig. 3), supports the possibility of chloroplast introgression from *J. regia* into *J. sigillata*.

Phylogenetic analysis revealed three highly supported clades (Maximum Likelihood Bootstrap Proportion (MBP) = 100, Posterior Probability [PP] = 1) in both the maximum likelihood (ML) and Bayesian inference (BI) phylogenetic trees (Fig. 5a, b). These clades comprised the outgroups, the *J. sigillata* clade and the *J. regia* clade, with the latter comprising two sub-clades (H1−H4 and H5−H11). No relationships within sub-clade H1−H11 had any statistical support, mirroring the star-shaped pattern in the network analysis (Fig. 5). The most common shared haplotypes, H1 and H5, were located within subclades H1–H4 and H5–H11, respectively, with both subclades also containing private haplotypes.

Principal component analysis (PCA) of the chloroplast genomes revealed a clear separation between *J. regia* and *J. sigillata* along the first principal component (PCA1, explaining 86.61% of the variance) (Fig. 5b). *J. regia* was predominantly represented by a single high-frequency haplotype (H5; 521 samples), whereas *J. sigillata* was primarily associated with haplotype H12 (42 samples). The PCA2 further subdivided the *J. regia* clade into two subgroups, corresponding to haplotypes H1−H4 and H5−H11. Overall, the PCA clustering was consistent with the network and phylogenetic structure of haplotypes (Fig. 5b).

#### Spatial distribution of haplotypes

The geographic distribution of haplotypes provides further insight into the history of cultivation. Geographically, the seven haplotypes of the *J. sigillata* clade (H12–H18) were restricted to western Yunnan and southeastern Tibet, comprising 61 individuals (Fig. 5d). In contrast, the 11 haplotypes of the *J. regia* clade (H1−H11) spanned southwestern to northern China. Of these haplotypes, nine were private, among which seven were distributed in northern China, whereas H3 and H4 were unique to southeastern Tibet (Fig. 5d). Haplotypes from the *J. regia* clade occurred within all *J. regia* individuals, some hybrids, and some *J. sigillata* individuals. The widespread shared haplotype H5, distributed throughout the sampling range, was present in 181 individuals of *J. regia*, 29 individuals of *J. sigillata*, and 311 individuals of hybrids, as defined by microsatellite data (Supplementary Table S2). The other common haplotype, H1, was found among 64 *J. regia* individuals and 61 individuals of hybrids. Haplotype H1, which likely evolved from H5, occurs more frequently in northern China than in southwestern China (Fig. 5d). These patterns reflect complex maternal lineage sharing, which may arise from historical movement, introgression, incomplete lineage sorting, or a combination of all.

### Maternal lineage bias in hybrids

Maternal lineage analysis based on cpDNA revealed a striking directional bias in introgression. Of the 1,011 hybrid individuals, only two individuals in southeastern Tibet possessed *J. sigillata* cpDNA, while the remaining 1,009 (99.8%, 95% CI: 99.2%–99.97%) across the hybrid zone inherited their chloroplasts from *J. regia*. This proportion is significantly different from a 50:50 expectation (binomial exact test, *p* < 0.0001), indicating that *J. regia* acts almost exclusively as the maternal parent in interspecific hybridization events.

### Integrating nuclear and chloroplast evidence

**Figure 6:**
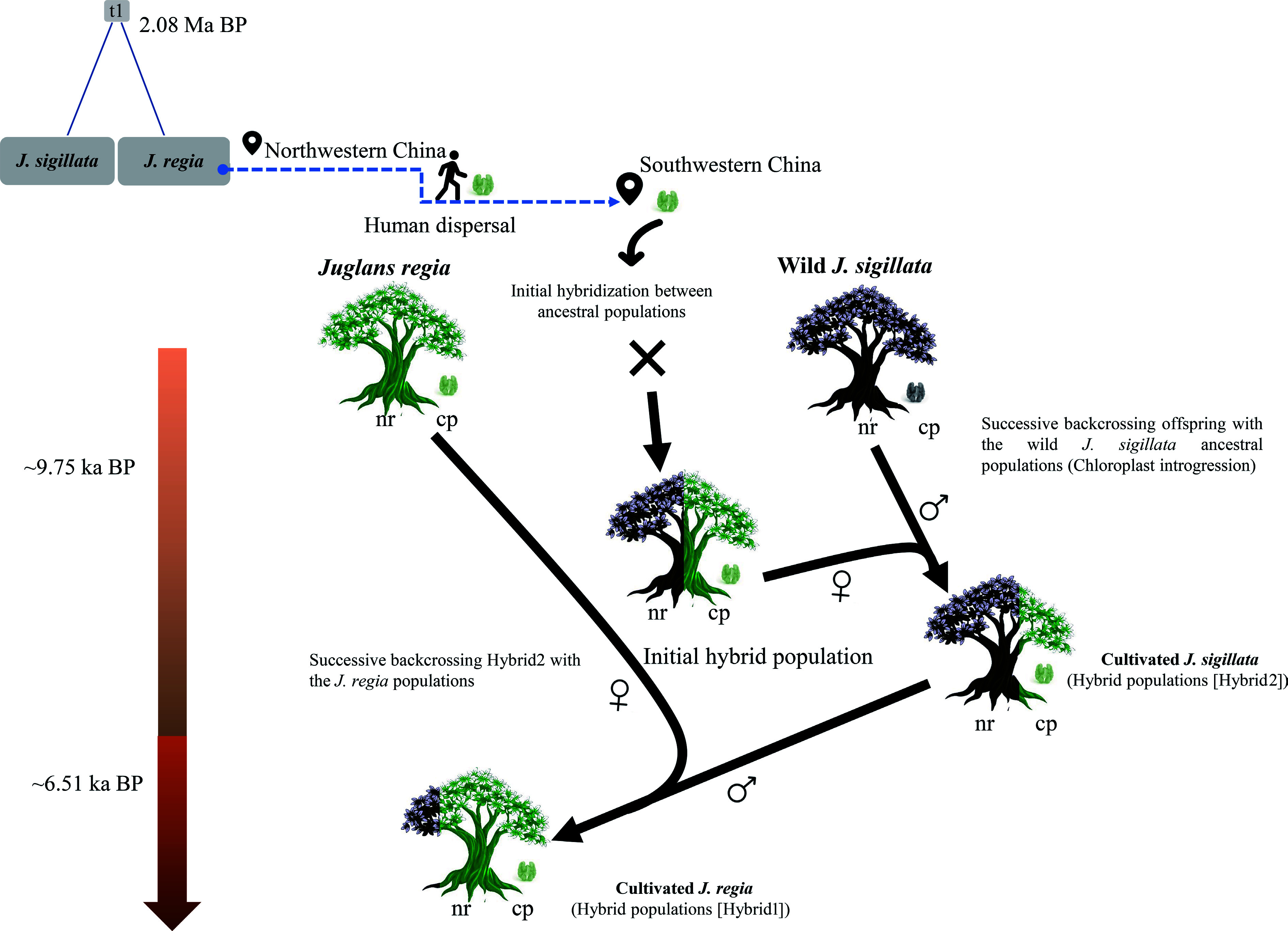
Schematical model for the hybrid origin of cultivated walnuts in southwestern China. *Juglans sigillata* and *J. regia* diverged approximately 2.08 million years ago. Briefly, during the Holocene, *J. regia* was spread to northern China and was later introduced into southwestern China following the emergence of cultivated hybrid walnuts there. The conceptual diagram depicts successive hybridization events and nuclear genome (nr) exchange, as shown by the color-coded tree stems representing nuclear genome composition. The chloroplast genome (cpDNA) is maternally inherited and is thus denoted by the female symbol (♀) to indicate its maternal origin, whereas the paternal parent is represented by the male symbol (♂). This model proposes the gradual chloroplast introgression in *J. sigillata*, driven by repeated genetic exchanges through hybridization. Notably, introgression is an ongoing process, not a discrete event.

The nuclear and chloroplast data present a clear but complex history of walnut hybridization (Fig. 6). The biparental nuclear microsatellites show that hybridization was a two-way process, creating the Hybrid1 and Hybrid2 groups with mixed ancestry from both species (Figs. 2–4). In contrast, the chloroplast genome tells a different part of the story. The maternally-inherited chloroplast genome is one of strong asymmetry; the vast majority of hybrids carry chloroplasts from *J. regia* (Fig. 5, Supplementary Table S2). The widespread *J. regia* H5 haplotype (Fig. 5d), found even in some pure *J. sigillata* underscores this pattern. So, while genes from both parents mixed freely in the hybrids, the maternal line was consistently dominated by *J. regia*.

## Discussion

### Drivers of walnut genetic diversity

Much of the cultivated walnuts in the study area of southwestern China comprises hybrids between *J. regia* and *J. sigillata*, including all examined populations that were cultivated as *J. sigillata*. According to various parameters, genetic diversity was higher in wild *J. sigillata* (JS) than the hybrids or *J. regia* (JR) (Table 1), consistent with Fan et al.^[32]^. Additionally, the greatest genetic differentiation (*F*_ST_) and genetic distance were observed between the JR and JS groups, while the hybrid group was positioned between them (Supplementary Fig. S3, Table 2). The unique geographical conditions and historical factors within the native range of *J. sigillata*, such as limited influence from Pleistocene glacial periods in southwestern China^[84−87]^, may have contributed to preserving population-level genetic variation. However, *J. sigillata* has experienced long-term human disturbance, which may have led to the elimination of some alleles, resulting in low genetic variability in the cultivated populations. Furthermore, low genetic variation in cultivated populations could be a consequence of artificial selection and/or a bottleneck due to breeding from a small number of genotypes^[88,89]^. A particular issue in this genus is that the extensive grafting of cultivars onto natural populations of *J. sigillata,* has led to rapid genetic erosion over the past three decades^[24]^. Conversely, founder effects following their ancient introductions of *J. regia* from the Western Himalaya via Central Asia^[23,32,46,47]^ might be responsible for the lower genetic diversity identified in *J. regia* populations in northern China. Notably, hybrids are discussed as a single genetic group in this context. The finer subdivision into Hybrid1 and Hybrid2 is applied specifically in the demographic analyses to interpret distinct hybridization histories.

Latitude was negatively correlated with genetic diversity based on microsatellite markers and latitude (Supplementary Fig. S2). Populations of *J. sigillata* (JS) and the hybrids groups had higher genetic diversity at lower latitudes. This genetic diversity gradient in walnuts likely reflects the impacts of cultivation, selection, and long-term agricultural practices such as the domestication and spread of walnuts across southwestern China. Therefore, although it meets the expectations of the Latitudinal Diversity Gradient (LDG) hypothesis, whereby biodiversity increases from the polar regions towards the equator^[90]^, the pattern in walnuts might be partially anthropogenic in its origin. Nonetheless, a correlation analysis suggests that environmental factors (Supplementary Fig. S2), which intensify with increasing latitude, may have contributed to the observed genetic variation gradient^[91,92]^ through natural selection. Therefore, the genetic differentiation observed in the latitudinal gradients of these populations likely results from a synergy of natural factors and human activities.

The highest genetic diversity was detected between 27° N and 30° N latitude, with prominent peaks at the western and eastern edges of southwestern China. Increased genetic differentiation at lower latitudes might result from limited gene flow in these regions^[93]^. In contrast, high genetic diversity in the western part of this region may be due to topographic and climatic factors^[94]^. Moreover, our findings indicated that the Three Parallel Rivers region harbors the highest genetic diversity (Fig. 3), which is consistent with previous meta-analyses of plant genetic diversity^[94,95]^. The region is known to have served as a Pleistocene glacial refugia for many taxa^[95−97]^. Because of the prolonged isolation and long-term persistence, populations in refugia are expected to retain higher genetic diversity^[98,99]^. Furthermore, the introduction of non-native *J. regia* germplasm and subsequent introgression between species may have enhanced regional genetic diversity. This further underscores the role of human activity in shaping genetic landscapes.

### Genetic landscape and origin of cultivated walnut

The population structure inferred from microsatellite data (Figs. 2, 3b) clearly distinguished the three groups (JR, JS, and Hybrid), with hybrids occupying an intermediate position while remaining clearly differentiated from both JR and JS. In contrast, chloroplast genome data (Fig. 5), reflecting maternal inheritance, revealed only two clades, corresponding to JR and JS. Within the JS group, seven haplotypes characterized by multiple mutational steps were identified, whereas the JR group contained 11 haplotypes separated by only a few mutational steps. Two haplotypes (H1 and H5) were dominant and widely distributed in JR, with the remaining haplotypes derived from H5. This topology forms a classic star-like phylogeny centered on H5, which strongly suggests a recent population expansion. These maternal lineage patterns are consistent with long-term persistence and isolation in *J. sigillata*, as opposed to a recent population expansion of *J. regia* in southwestern China, possibly reflecting anthropogenic dispersal.

Neighbor-joining analysis could not definitively determine whether the hybrid group was closer to the JR and JS groups (Fig. 2b, d). However, further analysis grouped populations into JR, JS, and two hybrid subgroups, Hybrid1 and Hybrid2, indicating their closer genetic affinity to JR and JS groups, respectively (Fig. 2d). Hybrid1 is morphologically similar to the JR group from northern China, while Hybrid2 consists of cultivated material morphologically identified as *J. sigillata* and commonly grown as soft-shell walnut (Chinese: 绵核桃, Mian Hetao). The JS group possessed far more private alleles (36) than the Hybrid (5) or JR groups (3). Likewise, JS scored highest on all other genetic diversity measures (Table 1). The low genetic diversity in both hybrid groups relative to JS (Supplementary Table S10) supports a scenario in which Hybrid1 and Hybrid2 originated from discrete hybridization events involving small effective population sizes (Fig. 6). This pattern is likely due to major geographic features, specifically, the mountains around Sichuan Basin and Yangtze River, which acted as significant barriers to north-south gene flow. Subsequent human-mediated dispersal likely facilitated the spread of these hybrids while reducing genetic diversity along dispersal routes. This further supports the view that JS represents long-standing wild populations, while JR and the hybrids appear to have been introduced more recently.

According to ABC analysis, *J. regia* and *J. sigillata* diverged around 2.08 Ma, during the early Pleistocene (Fig. 4). This estimate is broadly consistent with the Pleistocene divergence reported by Fan et al.^[32]^ (~1.41 Ma) and Ding et al.^[23]^ (~0.85 Ma). Although the estimates differ in timing, all studies confirm a critical period of diversification in the early to middle Pleistocene. The older divergence date in our analysis may be due to incomplete lineage sorting (ILS)^[100,101]^, undetected ancient gene flow^[102]^, or differences in sampling range^[103]^. A plausible explanation is that habitat fragmentation caused by Pleistocene climate oscillations promoted the divergence of *J. regia* and *J. sigillata*, likely in the Himalaya^[32,104]^. In the early Holocene, *J. regia* was likely first domesticated in the western Himalaya^[32]^, after which it spread to Europe, Central Asia, and northern China^[32,46,47,105]^.

Our results (Figs. 4, 5), consistent with previous studies^[23,48,106−108]^, indicate gene flow between *J. regia* and *J. sigillata* in southwestern China. Notably, we identified two hybrid groups that arose at different times (Fig. 4). The introduction of domesticated *J. regia* from northern China and its cultivation in southwestern China likely facilitated their hybridization, leading to the emergence of Hybrid2 around the early Holocene (~9.75 ka BP) (Fig. 6). Chloroplast genome data, reflecting strictly maternal inheritance, revealed that most hybrid individuals carried haplotypes common in *J. regia*, indicating that maternally inherited lineages from JR are common among hybrids. Our formal assessment of maternal lineage bias statistically confirms this predominance, demonstrating a significant asymmetry. The dominance of a limited number of these haplotypes, particularly H5, indicates that these specific maternal lines were preferentially selected and propagated, potentially reflecting their adaptive benefits. This systematic bias demonstrates directed human action, the repeated use of introduced *J. regia* as the seed parent, rather than a natural demographic process. Consequently, this pattern aligns with a domestication scenario in which superior *J. regia* varieties were served as maternal stock, and their hybrid progeny were subsequently spread across the region. Further intensification of human activity may have promoted further northward admixture between Hybrid2 and *J. regia*, yielding Hybrid1 by the middle Holocene (~6.51 ka BP) (Fig. 6). This suggests that the origin of cultivated walnut in this region predates the introduction of agriculture^[46,109]^, highlighting the influence of early human activities, such as hunting and gathering.

Several lines of evidence point to human-mediated dispersal as the primary driver for the walnut spread. First, a significant correlation was found between genetic variation and geographical distance for all populations and each group (Supplementary Fig. S2), suggesting that human movement, climate, and geophysical factors likely played a key role in shaping the genetic patterns^[103]^. Second, two haplotypes derived from *J. regia*, H5 and H1, were widely distributed the study region (Fig. 5). Most other haplotypes, many of which were private, were derived from H5 and differed by only one or a few mutational steps, often observed in single individuals, indicating a recent and rapid population expansion with insufficient time for substantial mutation accumulation. Third, although rodents contribute to walnut seed dispersal^[110]^, they are unlikely to account for the extensive and rapid dissemination of hybrids across heterogeneous terrain characterized by numerous mountains and rivers^[111]^. Finally, this genetic pattern is consistent with historical evidence: multiple waves of early human migration from the Yellow River basin into southwest China have been documented through both genetic and archaeological evidence^[112,113]^, with particularly intensified population movements occurring during the Qin (221–206 BC) and Han (206 BC–AD 220) dynasties^[114]^, including regions where Hybrid1 and Hybrid2 are found. Furthermore, historical trade routes in ancient China, such as the 'Tibetan-Yi Corridor' and the 'Southwest Silk Road', enabled the long-distance movement of humans and crops^[115−117]^. Collectively, our results indicate that large-scale human population movement may play a key role in the spread across southwestern China. The introgression of a few widespread *J. regia* haplotypes into diverse local *J. sigillata* populations provides a parsimonious explanation for the origin of cultivated walnut via human-mediated hybridization.

Haplotype H5 of the chloroplast genome was also present in many populations morphologically identified as *J. sigillata* (Fig. 5, Supplementary Table S2), consistent with additional contact and introgression between the two species, even where nuclear data suggest the samples as relatively pure JS (Fig. 2). The seven haplotypes originating from *J. sigillata* were confined to the westernmost part of its range. All private haplotypes were restricted to the eastern Himalaya. These populations occur in remote natural ecosystems, as confirmed by our field observations. Therefore, *J. regia* was likely brought south by humans, coming into contact with wild and/or cultivated *J. sigillata* and leading to the origin of multiple hybrid genotypes with varying parental contributions. Therefore, our data suggest a domestication history where human-introduced *J. regia* hybridized with local wild *J. sigillata*. This was not a random process. The frequent use of *J. regia* as the maternal parent, shown by the dominant H5 haplotype indicates a deliberate practice. We propose that people selectively cultivated and spread the hybrid offspring from these preferred *J. regia* trees, which ultimately formed the basis of the cultivated walnut germplasm known today as *J. sigillata* (Fig. 6). In essence, human-mediated introgression systematically transformed the local walnut population.

It is also important to consider the limits of our study. While our models point to specific times for divergence and hybridization, these are ultimately estimates based on the best-fitting scenarios, not precise dates. Furthermore, factors like incomplete lineage sorting or historical gene flow from populations we didn't sample could make the actual history more complex. Similarly, the genetic patterns we see fit very well with a story of human-mediated dispersal, especially the spread of a few walnut types across difficult terrain; it remains an inference based on patterns that are difficult to explain by natural dispersal alone. Finally, because we focused specifically on southwestern China, our findings might not fully unravel the entire history of these walnuts across their whole range. Despite these points, our study provides a powerful genetic framework for the origin of cultivated walnut, which can be tested more directly by future studies integrating ancient DNA, archaeology, and genomics.

## Conclusions

Using microsatellite markers and chloroplast genome data, this study examined the genetic diversity and population structure of 123 walnut populations to elucidate the evolutionary history of cultivated walnuts in southwestern China. Overall, our findings point to a history where natural landscape and human activity together shaped the genetics of walnuts in this region. The deep genetic split between *J. regia* and *J. sigillata* appears to have been maintained by the Sichuan Basin and the Yangtze River acting as long-term physical barriers. Their differing levels of diversity tell separate stories: *J. sigillata* likely persisted in glacial refugia, while *J. regia* shows signs of human-facilitated introduction. However, discordance between nuclear and chloroplast signals indicates that, despite the strong genetic division, gene flow has occurred since the Holocene, giving rise to two cultivated hybrid groups around the Sichuan Basin. Notably, *J. regia* appears to have contributed chloroplast genomes to the hybrids, as well as to some *J. sigillata* individuals. In the end, the patterns we see do not originate from just one process, but from the combined and sometimes contrasting effects of geography, climate history, and human activities. Future work will need more extensive geographic and genomic sampling to fully unravel the species' complex evolutionary history as well as the adaptive benefits and functional significance of gene exchange. Moving forward, efforts should focus on harnessing the superior germplasm hidden in natural mixed genetic pools, using an integrated genomic and ecological approach to bolster local adaptation and climate resilience.

## SUPPLEMENTARY DATA

Supplementary data to this article can be found online.

## Data Availability

All data generated or analyzed during this study are included in this published article and its supplementary information files.
