# Supplementary material for: Tracing the Holocene hybrid origin of cultivated walnut in southwestern China
Source: For Res (Fayettev). 2026 May 15;6:e018. doi: 10.48130/forres-0026-0018 (PMC13253123; doi:10.48130/forres-0026-0018)
Supplement: Supplementary file 1 — Supplementary data to this article can be found online. [file forres-0026-0018-S1.zip › 10.48130_forres-0026-0018-Suppl-FigureS1.pdf]

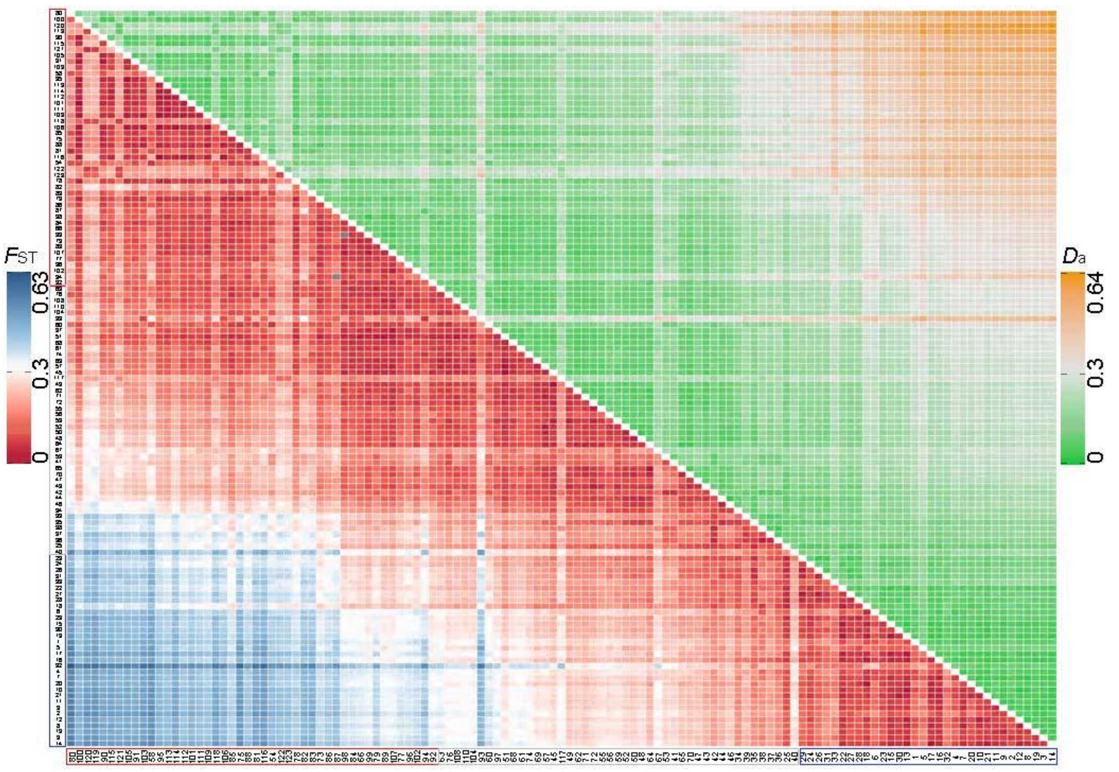

Fig. S1 Genetic differentiation (F_{ST}) and genetic distance (D_A) among 123 walnut populations. The lower-left section shows genetic differentiation (F_{ST}), while the upper-right section depicts Nei's genetic distance (D_A). The red, blue, and gray boxes along the lower and left margins represent *Juglans sigillata* (JS), *J. regia* (JR), and Hybrid groups, respectively.
