# Supplementary material for: Tracing the Holocene hybrid origin of cultivated walnut in southwestern China
Source: For Res (Fayettev). 2026 May 15;6:e018. doi: 10.48130/forres-0026-0018 (PMC13253123; doi:10.48130/forres-0026-0018)
Supplement: Supplementary file 1 — Supplementary data to this article can be found online. [file forres-0026-0018-S1.zip › 10.48130_forres-0026-0018-Suppl-FigureS2.pdf]

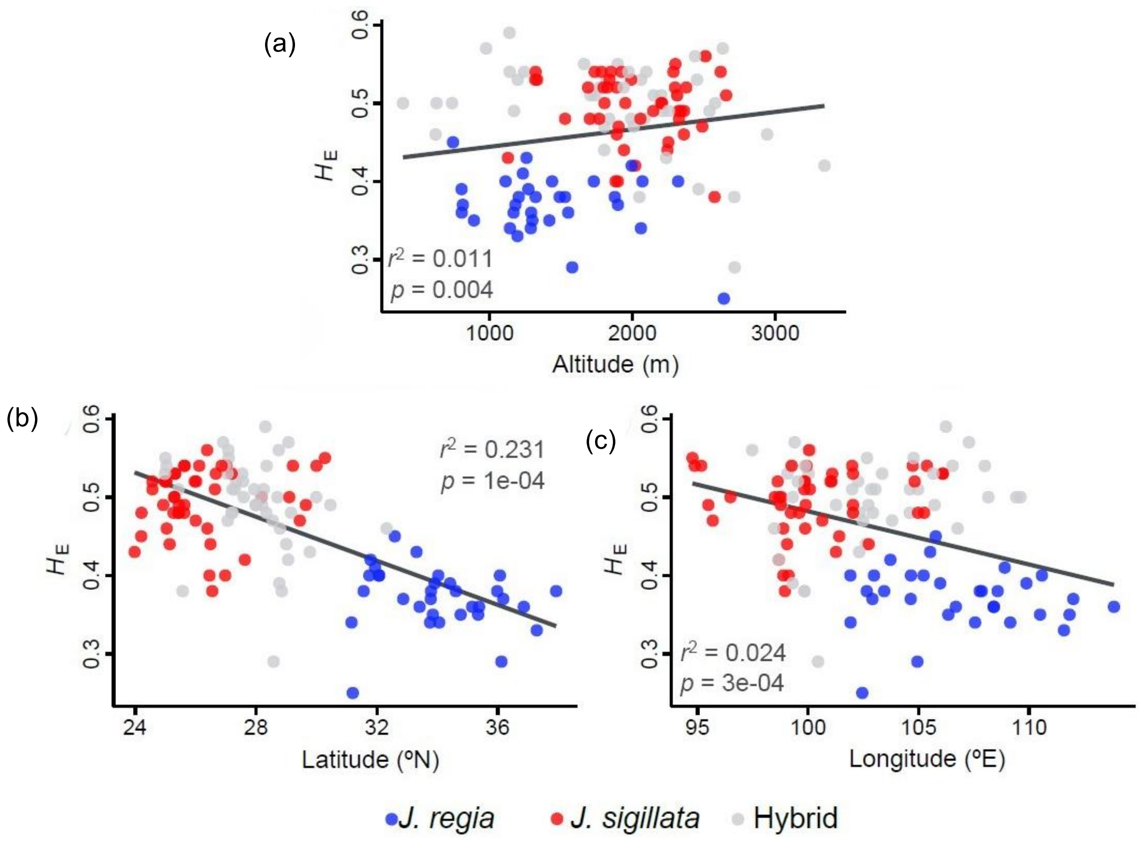

Fig. S2 Genetic differentiation and diversity in relation to geography. (a) Correlation between genetic diversity (H_E) and altitude; (b) Correlation between genetic diversity (H_E) and latitude; (c) Correlation between genetic diversity (H_E) and longitude.
