# Supplementary material for: Tracing the Holocene hybrid origin of cultivated walnut in southwestern China
Source: For Res (Fayettev). 2026 May 15;6:e018. doi: 10.48130/forres-0026-0018 (PMC13253123; doi:10.48130/forres-0026-0018)
Supplement: Supplementary file 1 — Supplementary data to this article can be found online. [file forres-0026-0018-S1.zip › 10.48130_forres-0026-0018-Suppl-FigureS3.pdf]

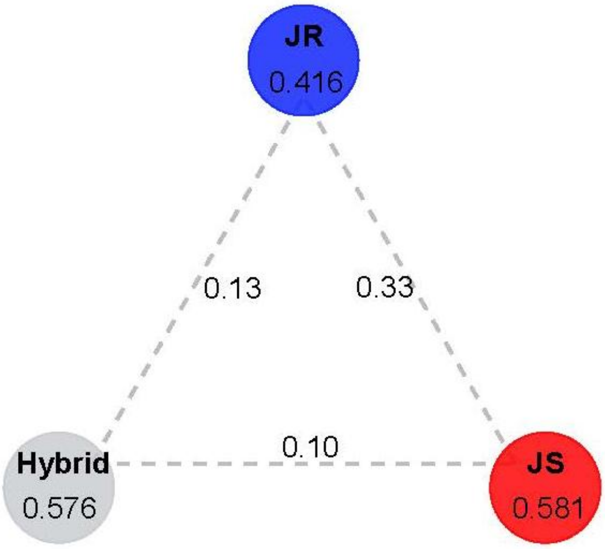

Fig. S3 Genetic diversity (H_E) and differentiation (F_{ST}) between various walnut groups in southwestern China. Values in circles indicate genetic diversity and values on each line indicate pairwise genetic differentiation.
