# Supplementary material for: Tracing the Holocene hybrid origin of cultivated walnut in southwestern China
Source: For Res (Fayettev). 2026 May 15;6:e018. doi: 10.48130/forres-0026-0018 (PMC13253123; doi:10.48130/forres-0026-0018)
Supplement: Supplementary file 1 — Supplementary data to this article can be found online. [file forres-0026-0018-S1.zip › 10.48130_forres-0026-0018-Suppl-FigureS4.pdf]

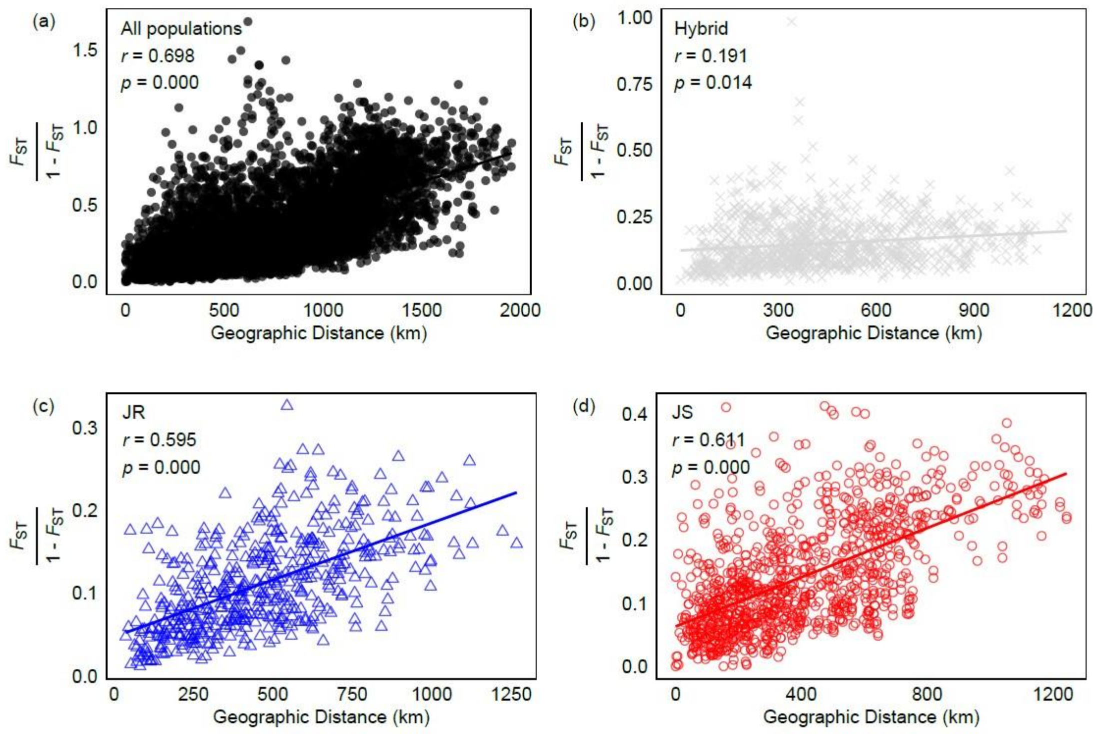

Fig. S4 Isolation-by-distance (IBD) analysis across population groups. Mantel tests correlating pairwise genetic distance (F_{ST}/1-F_{ST}) with pairwise geographic distance are shown for: (a) all populations combined, (b) hybrid populations only, (c) pure *J. regia* populations, and (d) pure *J. sigillata* populations.
